# Delineating the Intrinsic, Long-Term Path of Land Degradation: A Spatially Explicit Transition Matrix for Italy, 1960–2010

**DOI:** 10.3390/ijerph20032402

**Published:** 2023-01-29

**Authors:** Letizia Pace, Vito Imbrenda, Maria Lanfredi, Pavel Cudlín, Tiziana Simoniello, Luca Salvati, Rosa Coluzzi

**Affiliations:** 1 Institute of Methodologies for Environmental Analysis—Italian National Research Council (IMAA-CNR), c.da Santa Loja snc, I-85050 Tito Scalo, Italy; 2 Global Change Research Institute of the Czech Academy of Sciences, Lipová 9, CZ-370 05 České Budějovice, Czech Republic; 3 Department of Methods and Models for Economics, Territory and Finance (MEMOTEF), Faculty of Economics, Sapienza University of Rome, Via del Castro Laurenziano 9, I-00161 Rome, Italy

**Keywords:** degradation risk, ESAI, land trajectories, indicators, Mediterranean basin

## Abstract

Vulnerability to land degradation in southern Europe has increased substantially in the last decades because of climate and land-use change, soil deterioration, and rising human pressure. The present work focuses on a quantitative evaluation of changes over time in the level of vulnerability to land degradation of a Mediterranean country (Italy) using a composite indicator, the environmentally sensitive area index (ESAI), which is the final outcome of a complex model conceived to assess land vulnerability on the basis of climate, soil, vegetation, and human pressure. Considering four different levels of vulnerability to land degradation (not affected, potentially affected, fragile, and critical), the main trajectories of this index were highlighted in a long-time perspective (1960–2010), discriminating dynamics over two sub-periods (1960–1990 and 1990–2010). The empirical results at a very detailed spatial scale (1 km^2^ grid) reflect spatial consolidation of degradation hot-spots over time. However, aggregated trajectories of change indicate an overall improvement in the environmental conditions between 1990 and 2010 compared with what is observed during the first period (1960–1990). Worse environmental conditions concerned southern Italian regions with a dry climate and poor soil conditions in the first time interval, large parts of northern Italy, traditionally recognized as a wet and affluent agricultural region, experienced increasing levels of land vulnerability in the second time interval. Being classified as an unaffected region according with the Italian national action plan (NAP), the expansion of (originally sparse) degradation hot-spots in northern Italy, reflective of an overall increase in critical areas, suggests a substantial re-thinking of the Italian NAP. This may lead to a redesign of individual regional action plans (RAPs) implementing place-specific approaches and comprehensive measures to be adopted to mitigate land degradation.

## 1. Introduction

The third World Atlas of Desertification defined land degradation as a phenomenon leading “to a long-term failure to balance demand for and supply of ecosystem goods and services” [1,2]. Being a worldwide threat covering a multitude of socioeconomic and biophysical issues, land degradation implies several interacting impacts on soil quality and landscape structure and configuration, most of them induced by natural risks (e.g., drought, flood, landslide) on land, e.g., [3,4,5,6]. The encroachment of urban areas [7,8,9,10] and the improper use of soil and vegetation [11,12], however, remain important causes of land degradation in advanced economies.

The involvement of several dimensions underlying land degradation processes has stimulated a global debate as to how far the political dimension is concerned and represents a continuous challenge for scientists [13,14,15]. The synergistic effects of human drivers (whose impact reflects urban expansion, land-use intensification, tourism development, demographic growth, and depopulation, among others) and natural forces (poor soil features, scattered vegetation cover, rugged topography, climate aridity) contribute to the intrinsic complexity of the phenomena of land degradation. These factors, on the one hand, raise questions about the optimal methodologies identifying and monitoring affected areas in a comparable manner [16,17] and, on the other hand, stimulate a broader debate on the effectiveness of (direct and indirect) actions undertaken in any country of the world to fight desertification [18,19,20]. A part of this semantic and disciplinary complexity is linked to the range of socioeconomic, political, topographical, and climatic contexts impacted by land degradation, which affects the majority of land in all the continents of the world [21]. Just to mention some key figures, about one third of the total land area is regarded as degraded [22], with economic costs for recovery amounting to nearly 18–20 trillion USD per year (http://catalogue.unccd.int/856_Land_Based_Adaptation_ENG.pdf accessed on 12 December 2022).

The critical role of a healthy land in providing ecosystem services is known (https://www.eea.europa.eu/data-and-maps/indicators/land-take-3/assessment, accessed on 12 December 2022). Today, taking account of land-use/land cover and its (beneficial or detrimental) transformations over time becomes crucial with respect to the achievement of the strategic targets established by the United Nations (UN) through the SDGs—Sustainable Development Goals for 2030. This is particularly true for the specific target of land degradation neutrality (LDN, [23]) at the global level and by the EU through the European Green Deal at a continental scale [24]. In these contexts, the adoption of a system to ‘account’ environmental statistics in a harmonized, transparent, and easily interpretable manner is essential to pave the way toward a sustainable use of natural capital [25]. 

A specific focus on environmental degradation in Europe is justified with the fact that this continent—despite mostly affluent and technologically advanced—is not immune from desertification risk. About 25% of European land was classified at high or very high land degradation risk with a worrying trend of +14% with respect to the 2008 extent [17]. The northern Mediterranean Basin has long been identified as the most affected area within the continent [26]. A series of negative facts concern the high soil erosion rates [27], a low level of soil organic matter [28], the endemic problems of thin soils [29], the millenary artificialization of landscapes [30], and the high vulnerability to climate change due to rising temperatures, changes in seasonality rainfall patterns, and a particularly high frequency of extreme events (see, e.g., [31,32,33,34,35]). In such perspectives, Italy shares most of these negative historical and geophysical traits with other Mediterranean countries, maintaining, in turn, a strong (socioeconomic) gap between the northern and southern regions of the country [36,37,38].

Bearing in mind the urgency of this issue, linked to the idea of sustainability, assessment and monitoring of land degradation have been generally conducted by adopting indicators that provide synthetic information on the status and trends of the underlying degradation processes [39]. Indicators can be based on field measurements, socio-economic surveys or, if computed over large areas, on remote sensing investigations [3,40,41,42,43,44,45,46,47].

This indicator-based approach also includes the well-known environmentally sensitive area (ESA) methodology providing a comprehensive assessment of land vulnerability to degradation [48,49,50,51,52]. The Mediterranean Basin was the first area of its extensive application, and many studies demonstrate the reliability of this procedure when evaluating land vulnerability to degradation, since it considers multiple indicators related to four components: climate, soil, vegetation, and land management [53,54,55]. Research in the literature focused on multiple snapshots of the current conditions of land systems classified in terms of land vulnerability [56,57,58]. The typical approach adopted by ESA relies on geospatial data concerning land cover transitions whereas, in this work, we made the spatial transitions of land vulnerability classes completely explicit over a sufficiently long time span (1960–2010). Nevertheless, detailed investigations spanning a sufficiently long time period and estimating spatial trends of indicators have been rather occasional in Europe [59]. 

On the contrary, studies relying on the ESA procedure estimating subtle socioeconomic and ecological mechanisms that contribute to triggering land degradation [60,61] are aimed at capturing vulnerability changes over time from a vast set of indicators, revealing spatially unbalanced socioeconomic and environmental dynamics, e.g., [25]. This imbalance has inevitably reduced the effectiveness of policy actions contrasting land degradation in southern Europe. A new deal of strategies should be incorporated into the national framework to align Italy, and other Mediterranean countries, to Target 15.3 of the Sustainable Development Goals (SGDs), i.e., LDN by 2030 [22]. This consists of a broad corpus of local-based measures conceived to adapt to rapidly changing conditions and to curb land degradation risk, especially in areas classified as ‘historically not affected’ that, on the contrary, are recently experiencing a sharp worsening of environmental/ecological conditions, e.g., [62]. Based on these premises, the present work aimed to analyze the spatial distribution of different indicators computed at three points in time (1960, 1990, 2010) and delineate the evolution (i.e., change or stability) of land vulnerability levels over the Italian territory [63].

The analysis empirically estimates the transition probability of each elementary spatial unit from one class to another, allowing verification of the transition frequency both in a direction of a worsening of the boundary conditions with respect to desertification risk, and through trajectories of stability or improvement. This assessment has been carried out through raster-type spatial analysis methodologies, enabling the quantification of transition probabilities as a function of the different discrete classes defined ex ante. The obtained results can represent a useful tool to help policy makers in reaching (or, better, redefining) environmental targets [64].

## 2. Materials and Methods

### 2.1. Investigated Area

**Figure 1:**
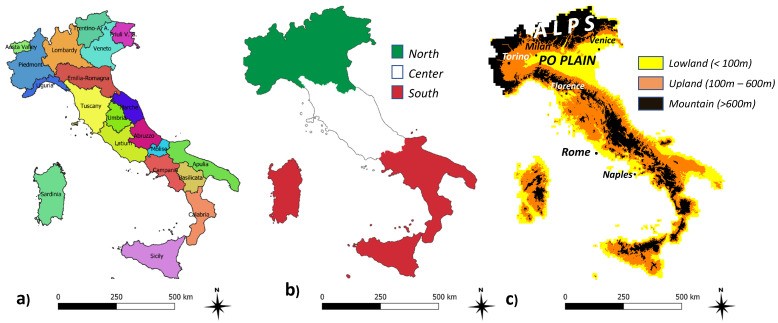
Partitions of Italy into: (**a**) 20 administrative regions according to nomenclature of territorial units for statistics (NUTS-2); (**b**) latitudinal belts; (**c**) elevation classes.

Italy covers an area of over 300,000 km^2^ in the heart of the Mediterranean Basin. Hills and mountains are the most common surfaces (42% and 35% of the national area, respectively) followed by plains (23%). Italy is considered an intriguing case for land degradation studies because of the asymmetric background concerning the three sub-areas in which the country is typically subdivided (north, centre, south [65]). First, a considerable socioeconomic gap exists among these three macro-regions [66,67,68] accompanied by different conditions concerning biophysical conditions of soil [69], climate [70,71], and vegetation quality [72]. This allows a refined analysis of the complex interplay between the socioeconomic and geo-environmental dimensions that contribute to determining the level of vulnerability to land degradation of a given territory [73]. In this context, the National Action Plan for Combating Drought and Desertification (NAP) was approved in 1999 and edited in accordance with the resolutions of the United Nations Assembly regarding the struggle against desertification, drought, and poverty. The Italian NAP identified southern Italy as an ‘affected’ region (Figure 1), whereas the northern and central regions of Italy were categorized as unexposed to severe land degradation [74].

### 2.2. Input Data and Variables in the ESA Model

In this section, a summary overview of the ESA framework with its relative data, variables and the final composite index (hereafter, the ESAI) is provided. The environmentally sensitive area (ESA) model, originally developed by Kosmas [75] within the MEDALUS project, is a suitable framework for desertification studies aimed at estimating the overall vulnerability level to degradation. Built up on a set of requirements affecting the reliability of the spatial outcome [76,77,78,79], the model is based on four pillars: climate, soil, land cover, and human pressure. To quantify the degree of vulnerability, a score system was used on the basis of the estimated level of correlation between each variable and land degradation [63,80,81].

Four indicators, containing information on land quality in terms of climate (climate quality index, CQI), soil (soil quality index, SQI), vegetation (vegetation quality index, VQI), and land management (land management quality index, MQI), were computed as the geometric mean of the different scores for each variable [82]. The scores of the ESAI ranged between 1 (the lowest vulnerability level) and 2 (the highest vulnerability level). We identified eight classes of vulnerability that can be optionally included in four macro-classes: not affected (NA), potentially affected (PA), fragile (F), and critical (C). The maps generated from this procedure have the spatial resolution of 1 km^2^ [83].

Among the four pillars, climate is a recognized factor of most land degradation processes [84,85]. The indicators of the CQI are based on data extracted from the National Agrometeorological Database of the Italian Ministry of Agriculture [14,73,86]. Different from climate, soil qualities are regarded as a quasi-static variable because their properties change slowly and, thus, soil is frequently considered a stable layer over time [65]. Soil data were extracted from various sources: (i) an Italian database of soil characteristics, (ii) a soil quality map produced by the European Desertification Information System for the Mediterranean (DISMED) project [87], (iii) ecopedological and geological maps of Italy, and (iv) a 20 m digital elevation model covering the overall national surface [85].

Starting from the Corine land cover database (CLC [88]), VQI reflects a vulnerability score system associated with each land cover on the basis of four variables: fire risk, protection against soil erosion, resistance of vegetation to drought, and plant cover [61,80]. Lastly, the MQI was built up, taking into account the impact of anthropogenic factors on land degradation through the evaluation of demographic and agricultural conditions (i.e., population density and trends, intensification of agricultural practices) using census data and CLC maps [25,63,81,89,90,91].

### 2.3. Procedure

**Figure 2:**
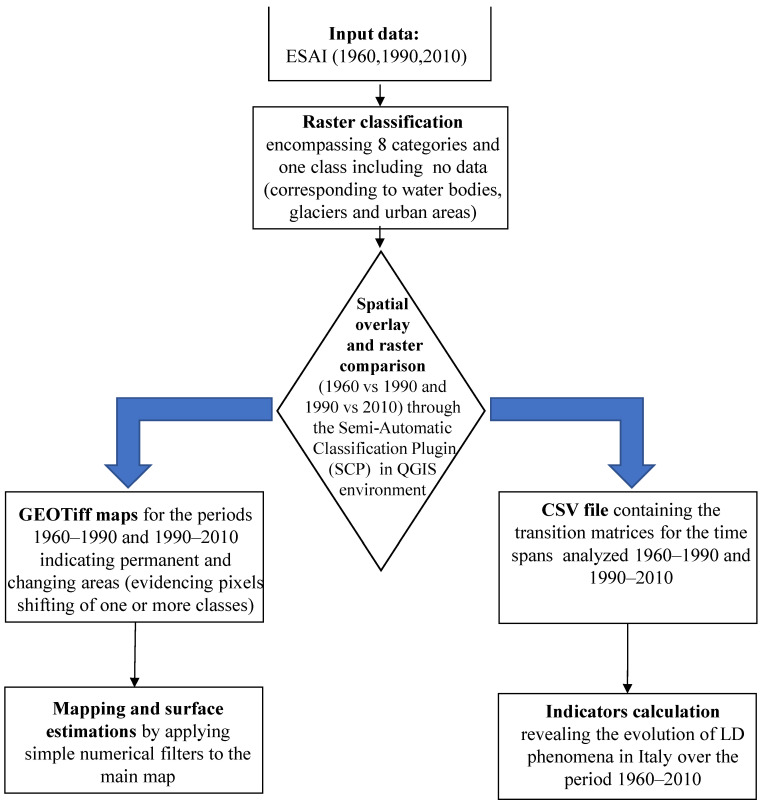
Flowchart of the adopted procedure.

Three ESAI rasters (dated 1960, 1990, and 2010) represent the input data of the geographic information system procedure aimed at extracting maps and relevant indicators of land degradation evolution in Italy between 1960 and 2010 (Figure 2). First, according to several authors [79,92,93], Italy was classified with a supervised segmentation of the ESAI into eight vulnerability classes. These account for not affected areas (NA), potentially affected areas (PA), fragile areas (including three classes with rising vulnerability levels: F1, F2, F3), and critical areas (including three classes with rising vulnerability levels: C1, C2, C3). Table 1 provides an overview of the main characteristics of each class. A ninth class was added to identify sealed areas, glaciers, and water bodies, which is useful to take account of possible changes occurring over time. Once the three rasters were prepared in a comparable way, we used the semi-automatic classification plugin (SCP, [94]) in the QGIS environment (version 3.16.6) to enable spatial overlay and raster comparison. This plugin was used to compare two rasters having the same number of classes that are labelled following a common criterion. The outputs of the SCP plugin are made up of two files: (i) a GEOTiff that visually enables the geography of occurred changes and (ii) a csv file reporting descriptive statistics related to land cover changes for each time window (1960–1990 and 1990–2010).

Each pixel of the obtained GEOTiffs shows values representing a specific combination between the two classifications, taking into account both stable and changing classes. For instance, all the pixels moving from class 4 to class 1 in the transition 1960–1990 (i.e., from F2 to NA) will have the value 7, whereas all the pixels moving from class 5 to class 1 (from F3 to NA) will have the value 11, and so on. Once the GEOTiff file is rendered, land cover change statistics appear in the ‘Output Tab’, making it simple to distinguish each class change and giving information about the extent of stable and shifting areas (km^2^). For each time span, the csv file includes the transition matrix from which it is possible to extract relevant indicators assessing the evolution of land degradation in Italy between 1960 and 1990 and between 1990 and 2010. These indicators, along with the single classes, can be displayed by applying a simple mask on the main GEOTiff file.

## 3. Results

**Figure 3:**
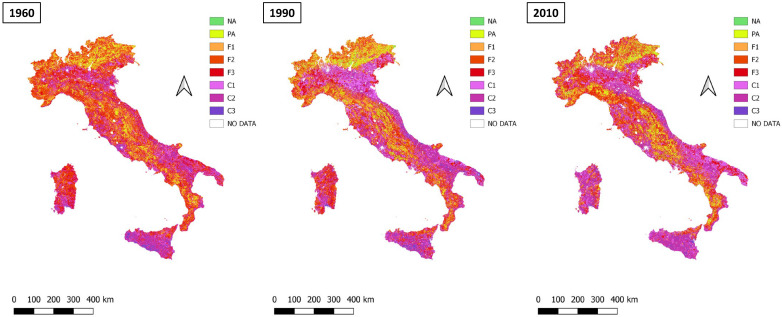
Classification of the Italian land according to the eight vulnerability classes for the years 1960, 1990, and 2010.

Patterns of land vulnerability, relying on the ESAI, are shown in Figure 3 for three time points (1960, 1990, 2010) with the corresponding surface area (Table 2).

Non-vulnerable areas (NA+PA) expand over time (from 5% to about 9.5% of the total landscape); the main transformations, however, concern the decrease in fragile areas occupying almost two-thirds of the whole Italian territory in 1960 and reduced to less than half of the national surface in the subsequent time point (1990 and 2010). Table 3 reports a detailed vulnerability accounting after classification of the study area into eight vulnerability categories.

**Figure 4:**
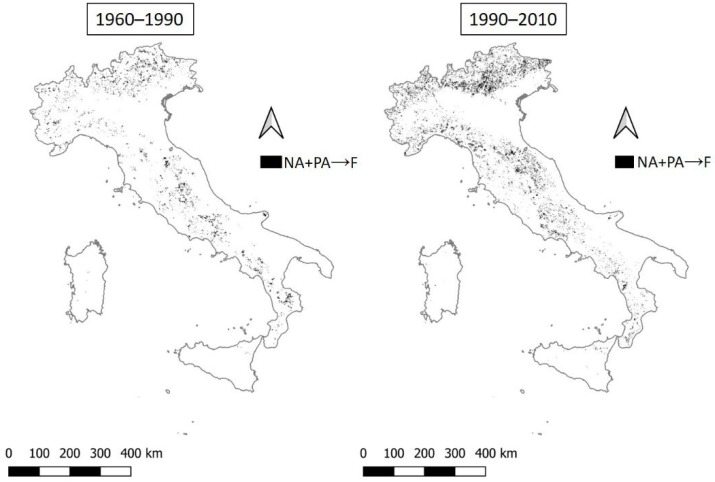
The transition from not affected or potentially affected land to fragile areas (NA+PA→F) by time interval.

Subsequently, we identify the positive and negative transitions observed in the two timeframes analyzed (1960–1990 and 1990–2010, see Table 3). The most relevant transition is the expansion of areas with higher vulnerability (from unaffected or potentially affected to fragile land, Figure 4), concerning areas that are not considered vulnerable and that pass to a state of initial land fragility. This is the main transition observed in both time periods. Such a transition has a rather heterogeneous spatial distribution over time, with an extension that doubles between 1990 and 2010 compared with the previous time interval (from 2.5% to 5.3%, see Table 4). This land transition is concentrated in northern and central Italy, especially in mountainous Apennine and upland contexts. In these areas, dedicated monitoring efforts seem to be required.

**Figure 5:**
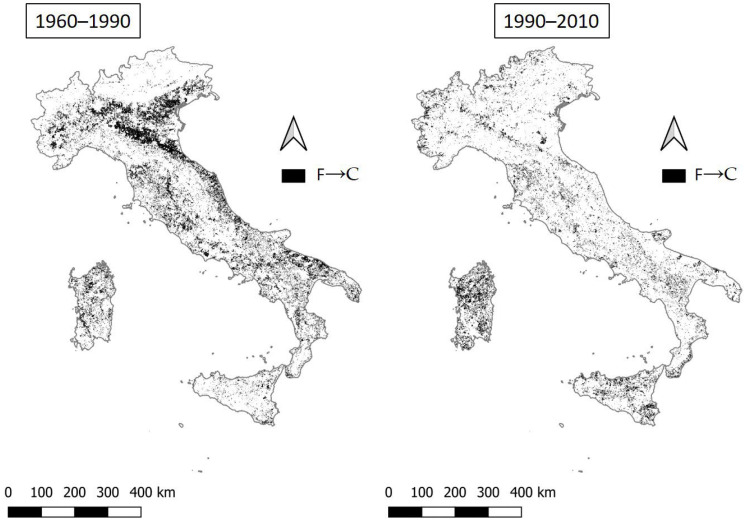
The transition from fragile to critical areas (F→C) in the two time intervals 1960–1990 and 1990–2010.

The remaining transition (worse conditions) is from fragile to critical areas (Figure 5). This step is very evident between 1960 and 1990 and less evident in more recent times, meaning that the expansion of critical areas is fundamentally associated with pre-1990 dynamics and concentrated in already fragile areas, namely, those including northern plains, flat and hilly areas of central Italy, and, above all, coastal areas. In southern Italy, this transition between 1960 and 1990 is revealed to be equally striking. In general, this transition is less evident between 1990 and 2010, and mainly concerns Sicily and Sardinia and both coastal and hilly-mountain territories in the rest of Italy. In northern Italy, this process seems to be less widespread in the last period than in southern Italy. In general, the phenomenon slows over time, with an area that has dropped from 18.6% to 11.3% when comparing the first with the second period (Table 4). The transition from fragile to critical land highlights traditional degradation dynamics, strongly associated with rising human pressure and loss of healthy vegetation cover, being, in turn, less dependent on recent climatic dynamics.

**Figure 6:**
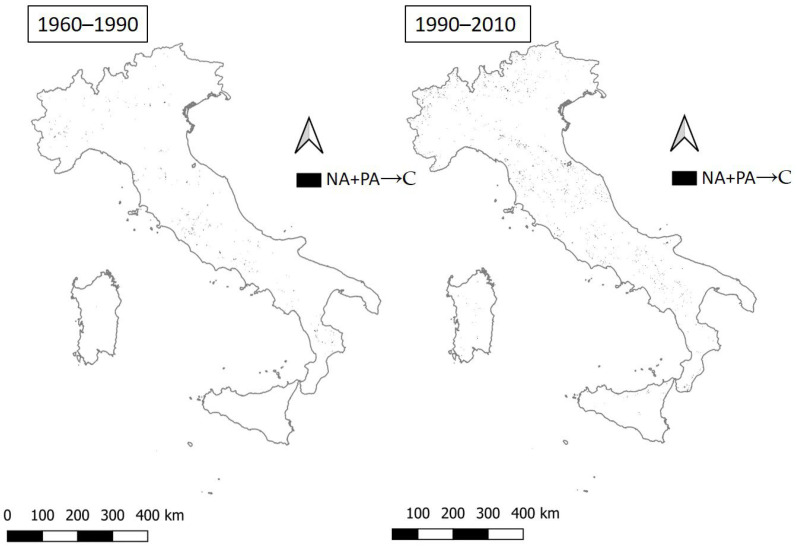
The transition from NA+PA to critical areas in the two time intervals 1960–1990 and 1990–2010.

For both periods, the transition from non-vulnerable areas to critical areas is very rare, featuring a jump of two classes, which represents a significant worsening of environmental conditions (Figure 6). Worse conditions predisposed to land degradation are more scattered across the national territory, consolidating hot-spots of variable size, namely, few pixels of a few square kilometers, experiencing a sort of ‘pulsation’. Reversing this pattern implies policies that cannot act exclusively on a national or regional scale, but requires monitoring strategies that increasingly focus on the local scale. 

**Figure 7:**
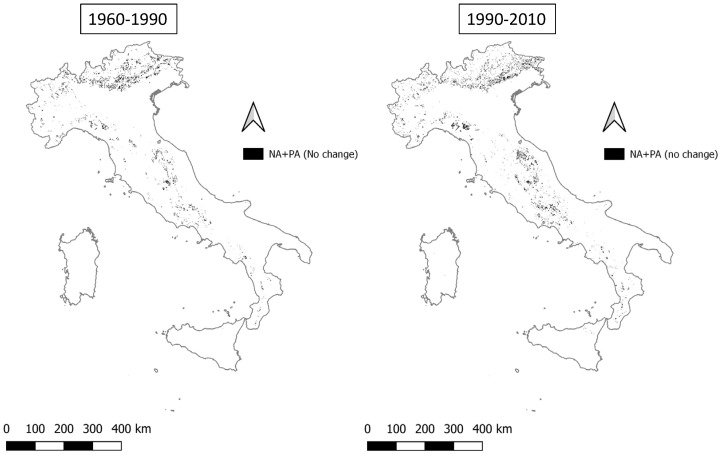
Areas belonging to not affected and potentially affected classes that remain stable (NA+PA (no change)) in the two time intervals 1960–1990 and 1990–2010.

The stability of the less vulnerable classes, meaning the unaffected or potentially affected classes, is mainly concentrated in Alpine and Apennine territories (Figure 7), suggesting that these areas can be considered as buffer zones that strongly maintain the structure of the territory and its characteristics of good quality, in terms of climate, vegetation, and land use. These areas amount to 1.96% in the period 1960–1990 and grow to 2.45% in the period 1990–2010 (Table 4).

**Figure 8:**
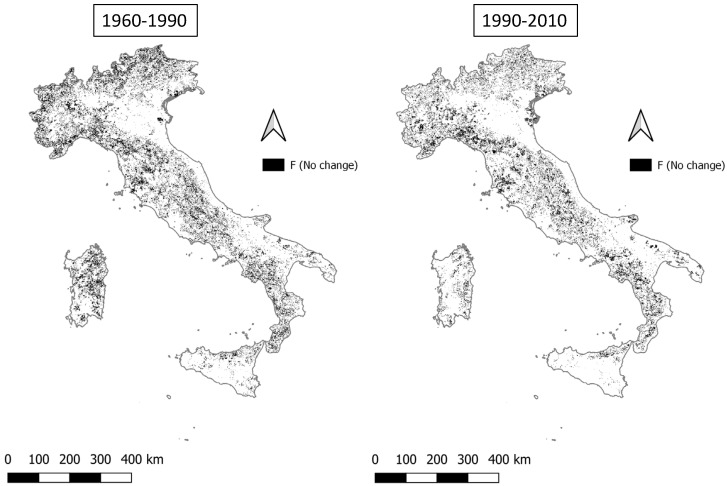
Fragile areas remaining stable (F (no change)) in the two time intervals (1960–1990 and 1990–2010).

Areas remaining stably in the ‘fragile’ (F) macro-class between 1960 and 1990 (17.7% of total landscape) are distributed over the entire national territory, excluding the Po Valley, southern Sicily, and Apulia (Figure 8). In the subsequent period, the same category shows a decrease to 13.6% and a greater concentration of clusters in mountainous territories.

**Figure 9:**
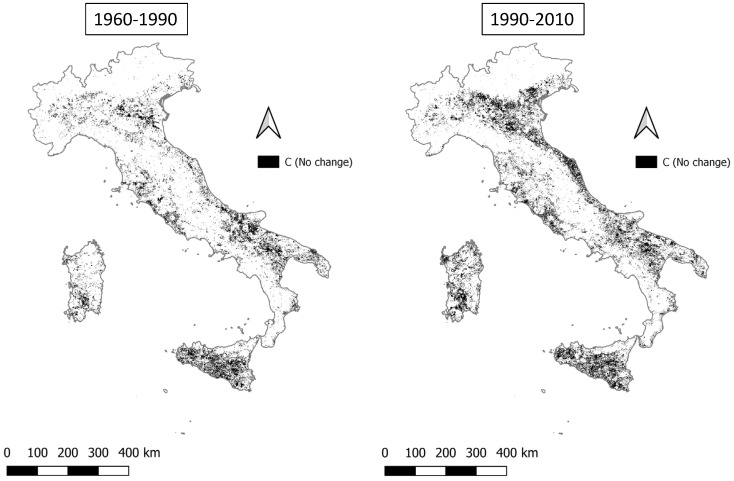
Critical areas remaining stable (C (no change)) in the two time intervals (1960–1990 and 1990–2010).

The percentage of land stably classified as ‘critical’ (C) increases from 12.4% (1960–1990) to 17.2% (1990–2010). In both periods, areas classified in this macro-group are distributed mostly along coastal and flat areas (Figure 9).

**Figure 10:**
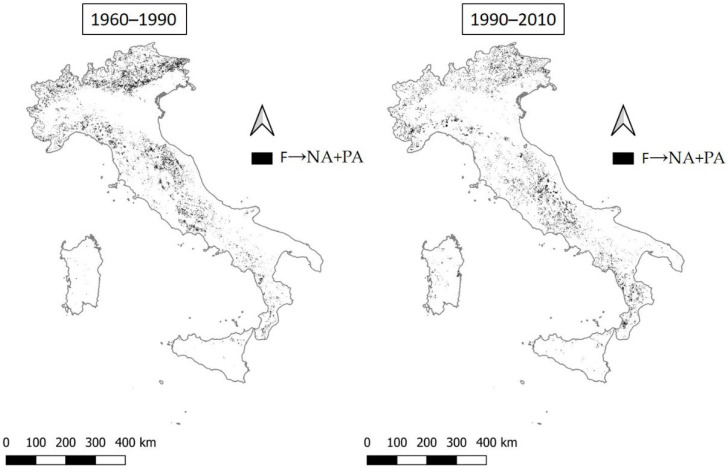
The transition from fragile areas to not affected or potentially affected areas (F→NA + PA) in the two time intervals 1960–1990 and 1990–2010.

The switch from the fragile macro class (F) to the potentially affected class (PA) stands at 5.6% for the period 1960–1990 and decreases to 4.7% in the period 1990–2010 (Figure 10). The areas gaining a lower vulnerability are mainly mountainous, belonging to the overall Alpine arch and Apennine chain.

**Figure 11:**
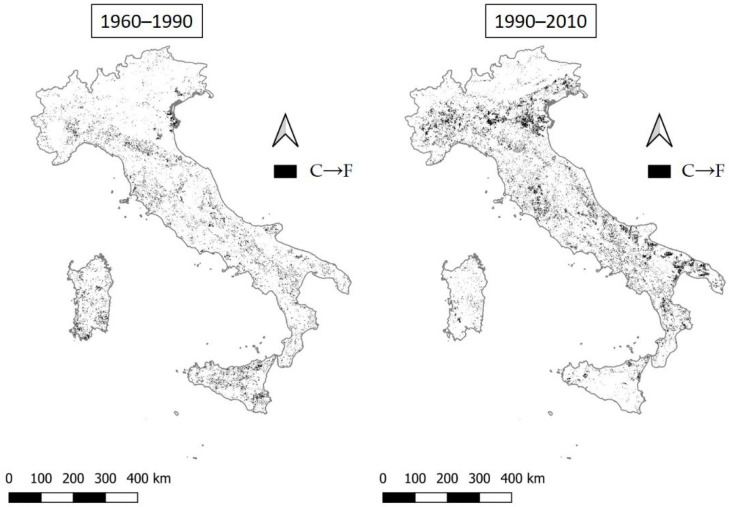
The transition from critical to fragile areas (C→F) in the two time intervals 1960–1990 and 1990–2010.

In the first period (1960–1990), the percentage of critical areas (C) shifting to fragile areas (F) is 6.6% and is concentrated in central and southern Italy, including major islands (Figure 11). The same transition in the second period (1990–2010) increases to 10.2%, with a countrywide distribution of the phenomenon and major clusters found in the Po Valley and Apulia.

**Figure 12:**
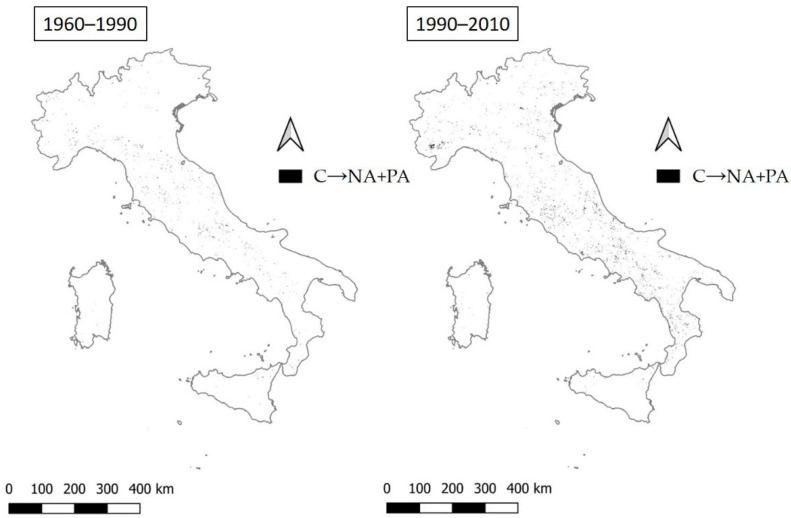
The transition from critical to not affected and potentially vulnerable areas (C→NA+PA) in the two time intervals (1960–1990 and 1990–2010).

Compared to what is reported for the transitions NA+PA→C, the positive change for C→NA+PA consists of scattered locations widespread in Italy that do not feature a clear geographical pattern (Figure 12). In the second period (1990–2010), their number increases, meaning overall better environmental conditions.

## 4. Discussion

This work assumes that the risk of desertification on a local scale in Italy takes place as an intrinsic property of local systems that evolve according to complex (and frequently non-linear) trajectories, and which are subject to the action of multiple drivers of change [12]. In this context, we adopted a composite index, the ESAI, to monitor land degradation considering four relevant dimensions, namely, climate, soil, vegetation, and human pressure [61]. Quality indicators allow for a multidimensional and integrated assessment of land vulnerability, evaluating local contexts according to multifaceted criteria integrating environmental, social, and economic dimensions of sustainability (e.g., [9]). Based on this information, a trade-off between sustainability and resilience of local systems can be outlined, whose long-term development is altered by exogenous shocks that limit or modify their evolutionary path [95].

Desertification risk is one of these exogenous shocks, being associated with worse conditions of land degradation [18]. With this perspective in mind, our analysis developed a descriptive procedure reflecting the operational philosophy of transition matrices (and, more generally, the idea underlying any change detection analysis), estimating the frequency of change from one state to another, which can be assumed as a dynamic property of complex local systems [96]. States were modelled in this study considering four vulnerability macro-classes, which express a gradient of land vulnerability [16]. Validity and internal coherence of these classes, as a function of the increasing level of land vulnerability, were verified both theoretically and through field work [76,97]. Classes were recognized as appropriately conceived to delineate local conditions at a strongly disaggregated spatial scale, such as the one used in our work [48]. Based on this background, the empirical analysis estimates the transition probability of each elementary spatial unit from one class to another, allowing a full comparison of transition frequencies (i.e., from a given vulnerability state to another) with stable trajectories over time [37]. These evaluations were developed adopting a raster-type spatial analysis that quantified transition probabilities as a function of a number of discrete classes fixed ex ante. The empirical analysis of the transition probabilities from one class to another is considered a preparatory tool for the construction of refined risk models from both probabilistic and non-linear perspectives, mimicking long-term dynamics of local systems [98]. 

Our study analyses a long-time horizon (from 1960 to 2010) with a subdivision in two intermediate time intervals (1960–1990 and 1990–2010). This timing enables the analysis of short-term dynamics in the evolution of desertification risk on a national and local scale, evidencing the inherent consolidation of processes of spatial convergence (or divergence) over time [65]. Spatial convergence implies a consolidated trend towards worsening or improved conditions of land vulnerability [99], being important for local policies, and allowing the delineation of boundaries of the so-called hot-spots [100]. On these areas, it is appropriate to intervene with formal policy mechanisms, or, conversely, they could represent examples of direct or indirect good practices for the containment of land degradation [21], e.g., areas that respond to the demands and targets of the LDN [101].

On the basis of the empirical results presented in this work, further research is needed as regards the creation of reliable, spatially explicit, and temporally structured risk indicators, allowing for a refined forecasting of desertification risk that local systems undergo as a function of multivariate drivers [53]. From this perspective, risk indicators can be calculated adopting both empirical and theoretical perspectives [12]. Risk frequency is associated with the marginal elements of the statistical distributions that have shaped this process. For instance, Markov chains, with a change of state that indicates greater or lesser risk than the starting value, allow a stochastic modelling of these environmental processes, both on a raster (pixel) basis and on a vector (polygon) basis. While the pixel-based analysis helps in reducing some quantitative problems, such as the effect of territorial partitions (the so-called modifiable areal unit problem (MAUP) [102]), the use of empirical or theoretical risk indicators on a polygonal scale (or administrative boundaries) allows for a more immediate application to spatial planning.

## 5. Conclusions

Land degradation is no longer associated exclusively with biophysical factors, such as climate and vegetation loss, being increasingly dependent on the rising human pressure on soil quality. The methodology here proposed highlights different spatial patterns of land vulnerability between 1960–1990 and 1990–2010. These patterns can be traced back to the effects of different land degradation drivers that have acted differentially in space and time. Between 1960 and 1990, worse conditions predisposed to land degradation involved areas already degraded or partially degraded. Between 1990 and 2010, the geographical gradients associated with worse conditions predisposed to land degradation were of socioeconomic relevance. Basically, land vulnerability was associated with spatial heterogeneity, implicit in the ESAI model and hardly attributable to traditional elevation (or urban–rural) gradients. In this sense, results suggest how policies should move further away from the dashboard of national guidelines prescribed in the Italian national action plan, which should be updated, similar to other Mediterranean countries with comparable socioeconomic and environmental features. The revision of regional and local plans fighting against desertification should accompany a through rethinking of the national action plan, definitely delineating appropriate and efficient application scales in terms of governance. This implies a new governance addressing the premises of zero net land degradation strategy (ZNLD). The objective of ZNLD should be not exclusively achieved on a national scale, focusing instead on the specificity of local territories and land degradation hot-spots. For instance, land experiencing an overall improvement in the local conditions predisposed to land degradation represents candidate examples of (formal or informal) good practices to fight against desertification. Future research should increasingly focus on factors related to the improvement in local conditions predisposed to land degradation and the underlying socio-ecological dynamics.

## Figures and Tables

**Table 1:** Characterization of the eight classes segmenting the ESAI.

ESAI Class	ESAI Score	Vulnerability Class	Area Description
Not affected	<1.17	Not affected	Non-threatened areas
Potentially affected	1.17–1.22	Potentially affected	Low vulnerability areas to be considered at risk only in the case of a severe worsening of climate and land management conditions.
Fragile 1	1.23–1.26	Fragile	Medium vulnerability areas in which any change altering the balance between natural and anthropogenic activities (e.g., climate change, occurrence of natural disaster, land use/cover changes) can trigger land degradation.
Fragile 2	1.27–1.32		
Fragile 3	1.33–1.37		
Critical 1	1.38–1.41	Critical	Degraded areas (e.g., sparse vegetated zones characterized by erosional processes) influencing surrounding areas.
Critical 2	1.42–1.53		
Critical 3	>1.53		

**Table 2:** Spatial partition of land vulnerability macro-classes by year.

Vulnerability Class	2010 (%)	1990 (%)	1960 (%)
Not affected + potentially affected	9.468	9.048	5.002
Fragile	47.376	48.256	64.416
Critical	43.156	42.696	30.582
Total	100	100	100

**Table 3:** Partition of land vulnerability in eight classes for the time points 1960, 1990, and 2010.

Vulnerability Class	2010 (%)	1990 (%)	1960 (%)
Not affected	0.979	0.851	0.008
Potentially affected	8.488	8.197	4.994
Fragile 1	11.006	12.068	13.528
Fragile 2	18.940	19.932	29.796
Fragile 3	17.429	16.256	21.093
Critical 1	16.827	15.896	13.229
Critical 2	24.895	24.913	16.252
Critical 3	1.434	1.887	1.101
Total	100	100	100

**Table 4:** Land vulnerability trajectories (per cent share in total landscape) in Italy by time interval.

Class Transition	1960–1990 (%)	1990–2010 (%)
NA+PA→F	2.47	5.30
F→C	18.6	11.32
NA+PA→C	0.15	0.03
NA+PA (no change)	1.96	2.45
F (no change)	17.68	13.62
C (no change)	12.37	17.20
F→NA+PA	6.10	5.21
C→F	6.61	10.23
C→NA+PA	0.30	0.96

## Data Availability

The data presented in this study are available on request from the corresponding author.
